# Multi-omics prediction of swine production traits with linear mixed models

**DOI:** 10.1186/s12711-026-01100-6

**Published:** 2026-11-10

**Authors:** Levi Ayres, Henk Bovenhuis, Ioanna-Theoni Vourlaki, Miriam Piles, Maria G. Luigi-Sierra, Peter Karlskov-Mortensen, Mario P. L. Calus

**Affiliations:** 1 Animal Breeding and Genomics, Wageningen University & Research, Wageningen, The Netherlands; 2 Animal Breeding and Genetics, IRTA, Torre Marimon, Caldes de Montbui, Barcelona, Spain; 3 Department of Veterinary and Animal Sciences, Faculty of Health and Medical Sciences, University of Copenhagen, Copenhagen, Denmark

## Abstract

**Background:**

Current prediction of phenotypes often relies on single-nucleotide polymorphism (SNP) data from DNA microarrays. Advances in biotechnology now enable large-scale collection of complementary omics data, such as transcriptomics and epigenomics. How much these additional molecular layers improve prediction accuracy, compared to SNP-based models, merits investigation. We evaluated whether using RNA transcript abundance and DNA methylation levels from loin muscle biopsies increased phenotype prediction accuracies for four traits in swine: average daily gain (ADG), backfat thickness (BFT), residual feed intake (RFI), and strength score (STR). We obtained transcriptomic (RNA sequencing) and epigenomic (reduced representation bisulphite sequencing) data of 443 boars representing the Duroc, Landrace, and Yorkshire breeds. We fitted linear mixed models incorporating genomic, transcriptomic, and epigenomic effects, and evaluated predictive performance by the correlation between preadjusted and predicted phenotypes obtained through cross-validation.

**Results:**

When animals from all breeds were used, the predictive accuracy (measured by the correlation between predicted values and preadjusted phenotypes in leave-one-out cross-validation) for ADG ranged from 0.12 with genomic best linear unbiased prediction (GBLUP) to 0.37 with transcriptomic–methylomic BLUP (TMBLUP) and genomic–transcriptomic–methylomic BLUP (GTMBLUP). For BFT, accuracies ranged from 0.18 with methylomic BLUP (MBLUP) to 0.39 with genomic–transcriptomic BLUP (GTBLUP) and GTMBLUP. For RFI, accuracies ranged from 0.19 (pedigree BLUP, GBLUP, MBLUP) to 0.58 (GTBLUP). For STR, accuracies ranged from −0.27 (MBLUP) to 0.11 with transcriptomic BLUP (TBLUP), TMBLUP, GTBLUP, and GTMBLUP. Genomic–methylomic BLUP (GMBLUP) models exhibited intermediate predictive performances.

**Conclusions:**

Incorporating transcriptomic and epigenomic data from loin muscle biopsies increased the accuracy of phenotype prediction relative to standard GBLUP, with transcriptomic information serving as the primary driver of these gains. Among the single-omics models, TBLUP attained the highest accuracies, while MBLUP exhibited the lowest performance. The combined GTMBLUP model was among the best-performing models overall, although gains relative to GTBLUP or TMBLUP were modest when present.

**Supplementary Information:**

The online version contains supplementary material available at https://doi.org/10.1186/s12711-026-01100-6.

## Background

Genetic improvement of livestock populations has relied mostly on linear mixed model methodology and pedigree-based best linear unbiased prediction (BLUP) over the past five decades [1–5]. By modelling additive genetic effects through pedigree-derived relationship matrices, BLUP selection has helped deliver sustained genetic gains for complex production traits [6–8].

The availability of affordable DNA microarrays enabled genomic selection [9, 10], in which pedigree relationships are replaced or augmented by genomic relationship matrices (GRMs) [11–13]. Genomic BLUP (GBLUP) [14], or its equivalent BLUP of single-nucleotide polymorphism (SNP) effects (SNP-BLUP) [9, 15], preserves the classical mixed-model framework while improving the accuracy of estimated breeding values (EBVs) by capturing realized genetic relationships among individuals [16, 17]. As a result, genomic prediction has become a standard tool in modern livestock breeding programmes.

Advances in high-throughput sequencing have enabled the large-scale profiling of additional molecular layers (omics), including the transcriptome and the epigenome, at decreasing cost [18–20]. These data provide biological information beyond DNA sequence: the transcriptome reflects the functional state of the genome in cells, whereas the epigenome contributes to the regulation of gene expression and reflects aspects of developmental history. As such, these omics layers represent additional components of the genotype–phenotype map and may capture variation not explained by SNP markers alone, making them potential candidates for inclusion in prediction models, particularly for phenotype prediction. Unlike genotypes, omics measurements are not unequivocally defined: they depend on the tissue sampled, the assay used, and the time of sampling. Consequently, the contribution of omics information to trait prediction is context-dependent. A clear understanding of their predictive ability for different species, traits, and tissues is being consolidated [21–30].

From a statistical perspective, these omics can be incorporated into prediction models by constructing omics-specific relationship matrices and fitting them as additional random effects in a linear mixed model [27]. This enables the inclusion of multiple biological data sources while maintaining the computational and statistical advantages of classical BLUP approaches.

The objective of this study was to evaluate whether including transcriptomic (gene expression) and epigenomic (DNA methylation) information from longissimus muscle in linear mixed models improves the accuracy of phenotype prediction in boars from a commercial pig population. We compared single- and multi-omics models by assessing predictive performance as the correlation between preadjusted and predicted phenotypes. Furthermore, we estimated variance components to quantify the relative contribution of each omics layer to phenotypic variation.

## Methods

### Animals and phenotypes

We obtained data from the DanBred commercial breeding programme in Denmark. The dataset comprised records from 443 male, non-castrated pigs from three pure breeds: Duroc (143), Landrace (148), and Yorkshire (152). The animals were born in 2021–2022 and had phenotypes, pedigrees, genotypes, gene expression levels, and DNA methylation information available. Gene expression and DNA methylation levels were obtained by RNA sequencing (RNA-seq) and reduced representation bisulphite sequencing (RRBS), respectively, from longissimus dorsi muscle biopsies collected when the animals reached a body weight of approximately 100 kg.

We analysed four production traits: average daily gain (ADG), backfat thickness (BFT), residual feed intake (RFI), and strength score (STR)—a composite conformation trait. Traits were recorded over the testing period, which comprised the weight interval from approximately 30 to 100 kg of live body weight.

ADG (in grams per day) was measured over the testing period. Feed intake was recorded using electronic feeding stations. ADG was preadjusted within breeds for the following systematic environmental effects: section, sex, parity of dam, and body weight at the start of the testing period.

RFI (in grams per day) was defined as the residual from the linear regression of average feed intake on section, parity of dam, ADG, and metabolic body weight. Average feed intake was calculated as the total feed intake (in grams) during the testing period divided by the number of days in the testing period. An individual’s metabolic body weight (MBW) was defined as

1 $$\mathrm{MBW} = \frac{\mathrm{BW}_{\mathrm{t}_2}^{1.75} - \mathrm{BW}_{\mathrm{t}_1}^{1.75}}{1.75\left(\mathrm{BW}_{\mathrm{t}_2} - \mathrm{BW}_{\mathrm{t}_1}\right)},$$

where $\mathrm{BW}_{\mathrm{t}_1}$ and $\mathrm{BW}_{\mathrm{t}_2}$ denote body weight (in kilograms) at the beginning and end of the testing period, respectively.

Four backfat thickness measurements (in millimetres) were recorded per boar at the end of the testing period (approximately 100 kg body weight), and their means were used as phenotypes. BFT was preadjusted within breeds for section, sex, parity of dam, and body weight at the end of the testing period.

STR is a composite trait derived from the assessment of leg strength, back strength, and overall conformation. Each pig received an integer score from 1 to 14 at the end of the testing period. STR records were preadjusted for section, sex, parity of dam, and body weight at the end of the testing period.

### Information sources

The information sources considered for the estimation of variances and prediction of phenotypes were pedigrees, genotypes, DNA methylation levels (proportion of methylated reads), and gene expression levels (RNA transcript abundance). We used these data (pedigrees and omics) to construct relationship matrices and form the covariance structures of random effects in linear mixed models. We applied BLUP to predict phenotypes.

The single-omics models were GBLUP (Genotypes), MBLUP (DNA Methylation), and TBLUP (RNA Transcripts), while the multi-omics models were TMBLUP (Transcripts and Methylation), GMBLUP (Genotypes and Methylation), GTBLUP (Genotypes and Transcripts), and GTMBLUP (Genotypes, Transcripts, and Methylation). PBLUP (Pedigree) models were also fitted for baseline comparison. Furthermore, we estimated variance components associated with each information source to quantify their contributions to the total phenotypic variance.

#### Pedigrees

Three breed-specific pedigree files were used to construct the numerator relationship matrices of the PBLUP models. The Duroc, Landrace, and Yorkshire pedigrees contained 8942, 7587, and 6775 animals and spanned up to 44, 53, and 49 generations, respectively.

#### Genotypes

Animals were genotyped using the GeneSeek Genomic Profiler Porcine 50k SNP array (Neogen Corporation, Lansing, MI, USA). We filtered genotypes within each breed by excluding SNPs with a minor allele frequency less than 1.5%. After filtering, 24,656, 29,556, and 27,854 SNPs remained for the Duroc, Landrace, and Yorkshire breeds, respectively. Because the set of SNPs retained after filtering differed among breeds, we kept only SNPs retained in all three breeds for subsequent analyses, resulting in a common marker panel of 11,822 SNPs.

For the within-breed analyses, we constructed GRMs following VanRaden’s first method [14]:

2 $$\mathbf{G} = \frac{\mathbf{D}\mathbf{D}^{\top}}{2\sum \mathrm{p}_{\mathrm{i}}\left(1 - \mathrm{p}_{\mathrm{i}}\right)},$$

where **D** is the centred genotype matrix, in which twice the allele frequency of the corresponding locus is subtracted from each allele count, and p_i_ is the allele frequency at SNP i.

For the multi-breed analyses, we computed between-breed GRMs as [31]

3 $$\mathbf{G}_{\mathrm{bc}} = \frac{\mathbf{D}_{\mathrm{b}}\mathbf{D}_{\mathrm{c}}^{\top}}{\sqrt{2\sum\nolimits_{\mathrm{i}} \mathrm{p}_{\mathrm{b}_{\mathrm{i}}}\left(1 - \mathrm{p}_{\mathrm{b}_{\mathrm{i}}}\right)}\,\sqrt{2\sum\nolimits_{\mathrm{i}} \mathrm{p}_{\mathrm{c}_{\mathrm{i}}}\left(1 - \mathrm{p}_{\mathrm{c}_{\mathrm{i}}}\right)}}, \quad \mathrm{b} \ne \mathrm{c},$$

where **D**_b_ and **D**_c_ are centred genotype matrices for breeds b and c, and ${{\rm{p}}_{{{\rm{b}}_{\rm{i}}}}}$and ${{\rm{p}}_{{{\rm{c}}_{\rm{i}}}}}$are the corresponding allele frequencies at SNP i.

We then assembled the full multi-breed GRM in block form as [31]

4 $$\mathbf{G}_{\mathrm{all}} = \left\lbrack \begin{array}{ccc} \mathbf{G}_{\mathrm{DD}} & \mathbf{G}_{\mathrm{DL}} & \mathbf{G}_{\mathrm{DY}} \\ \mathbf{G}_{\mathrm{LD}} & \mathbf{G}_{\mathrm{LL}} & \mathbf{G}_{\mathrm{LY}} \\ \mathbf{G}_{\mathrm{YD}} & \mathbf{G}_{\mathrm{YL}} & \mathbf{G}_{\mathrm{YY}} \end{array} \right\rbrack$$

where diagonal blocks correspond to within-breed relationships and off-diagonal blocks correspond to between-breed relationships.

#### RNA transcripts

When pigs reached a body weight of approximately 100 kg, longissimus dorsi muscle biopsies were collected, snap-frozen in liquid nitrogen and stored at −80 °C until processing. The biopsies were used for RNA-seq and RRBS.

Samples with RNA integrity number greater than 6 were retained for polyadenylated messenger RNA (mRNA) library preparation and sequenced on an Illumina NovaSeq 6000 platform (Illumina, Inc., San Diego, CA, USA), generating 2 × 150 bp paired-end reads. The reads were quality-controlled, filtered, and aligned to the *Sus scrofa* reference genome assembly Sscrofa11.1 [32, 33]. More details about the laboratory protocols and bioinformatics methods employed can be found in Luigi-Sierra et al. [34].

RNA abundance values were expressed as counts per million (CPM). Lowly expressed genes were removed by retaining only those with CPM > 0.5 in at least 20% of individuals, resulting in an expression matrix containing 14,293 genes. To correct for compositional differences between libraries, trimmed mean of *M*-values (TMM) normalization was applied, followed by a logarithmic transformation of the normalized counts. All normalization and transformation steps were performed using the edgeR software package [35–37].

We computed the transcriptomic relationship matrix as

5 $$\mathbf{T} = \frac{\mathbf{R}\mathbf{R}^{\top}}{\mathrm{n}_{\mathrm{R}}},$$

where **R** is the matrix of transformed CPM values standardized per gene (centred to a mean of zero and scaled to unit variance) and n_R_ is the number of retained genes.

#### DNA methylation data

Genome-wide DNA methylation profiles of the longissimus muscle biopsies were generated for the male pigs from the three breeds using libraries prepared with the Diagenode Premium RRBS v2 kit (Diagenode SA, Liège, Belgium). The libraries were constructed following digestion with the enzyme MspI, adapter ligation with unique molecular identifiers (UMIs), bisulphite conversion, and polymerase chain reaction (PCR) amplification. Sequencing was performed on the Illumina NovaSeq 6000 platform to a mean coverage of approximately 40 × . For more details on library preparation and sequencing, see Luigi-Sierra et al. [34]. Raw sequencing reads were quality-controlled, trimmed, and aligned to the domestic pig reference genome Sscrofa11.1. UMIs were used for deduplication, methylation levels were quantified at cytosine–phosphate–guanine (CpG) dinucleotides, and contiguous CpG sites on opposite strands were merged. We obtained an initial dataset of approximately 4 million CpG sites. DNA methylation levels were quantified as proportions, obtained by dividing the number of methylated reads by the total number of reads at each CpG site.

CpG sites were restricted to autosomal regions; reads mapping to unplaced scaffolds, sex chromosomes, and mitochondrial DNA were excluded. We set observations with sequencing depth exceeding the 99.99th percentile of the coverage distribution (783× cutoff) as missing to avoid the influence of abnormally high coverage values likely due to technical artefacts. We retained a CpG site for downstream analyses if at least 90% of animals within each breed had a sequencing depth of 5× or greater at that site. We removed individual animals if more than 10% of their CpG observations across the retained sites were missing. The resulting dataset contained 443 animals and 1,095,304 CpG sites. We imputed all missing values in the filtered dataset with the breed-specific median methylation level at each CpG site, as downstream analyses required a complete methylation matrix. Furthermore, we imputed low-coverage observations (1–4 reads) through a Bayesian beta-binomial approach. To do this, we modelled the observed number of methylated reads as a binomial random variable with parameters *n* and *p*, where *n* is the read depth (1, 2, 3, or 4) and *p* is the underlying methylation proportion to be estimated. We used a beta distribution as the conjugate prior for *p*, with its parameters determined by the breed-specific median methylation level at each CpG site. The imputed value was the median of the resulting beta posterior distribution; if the posterior’s median was an extremely small numerical value (smaller than 10^−10^), we rounded it to zero. In total, approximately 2.7% of all CpG-level observations were imputed using this procedure.

**Fig. 1:**
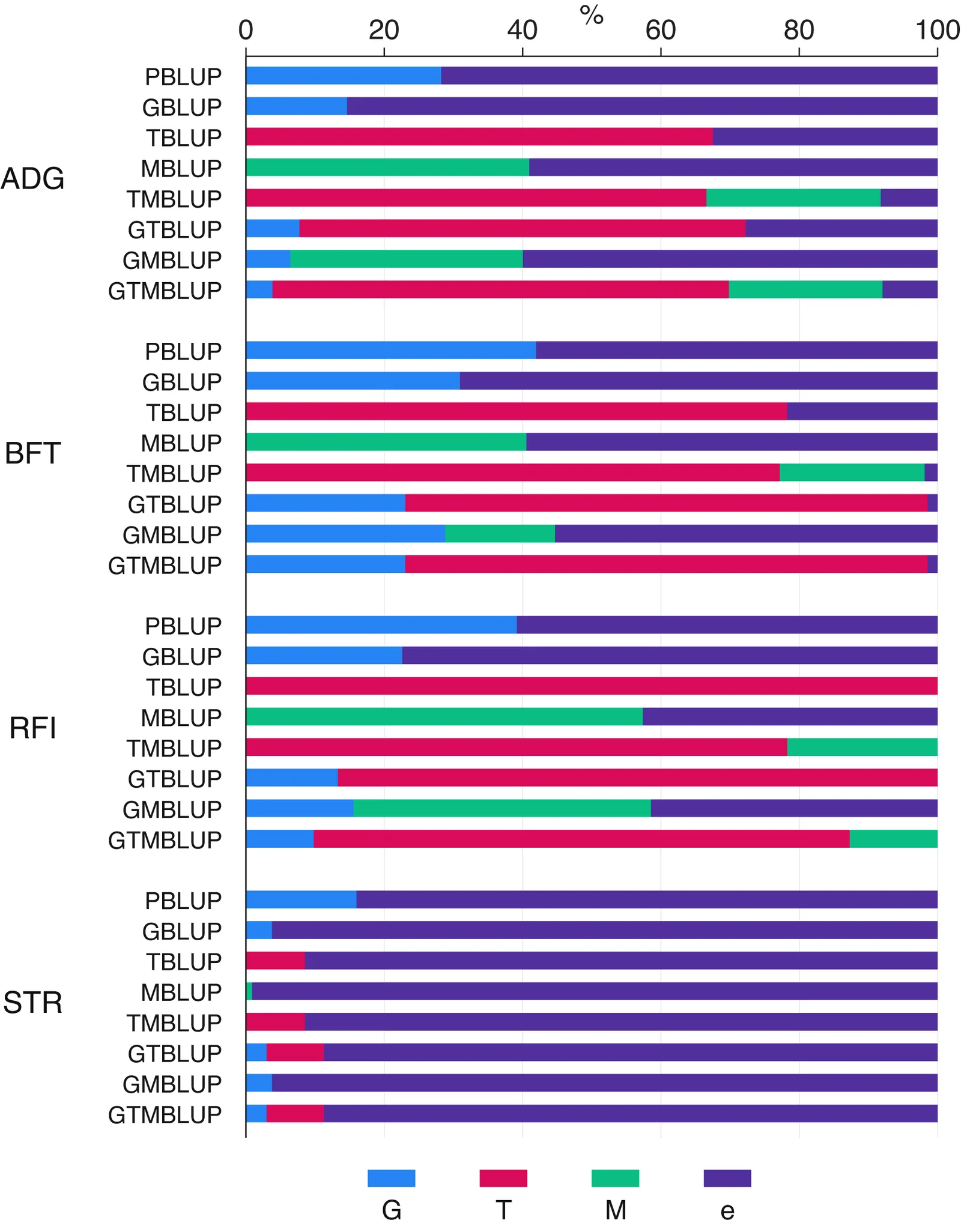
Variance components as proportions of total phenotypic variance. Coloured bar segments represent the proportions of phenotypic variance attributed to each component within a given model. G, genomic; T, transcriptomic; M, methylomic; e, residual. ADG, average daily gain; BFT, backfat thickness; RFI, residual feed intake; STR, strength score

**Fig. 2:**
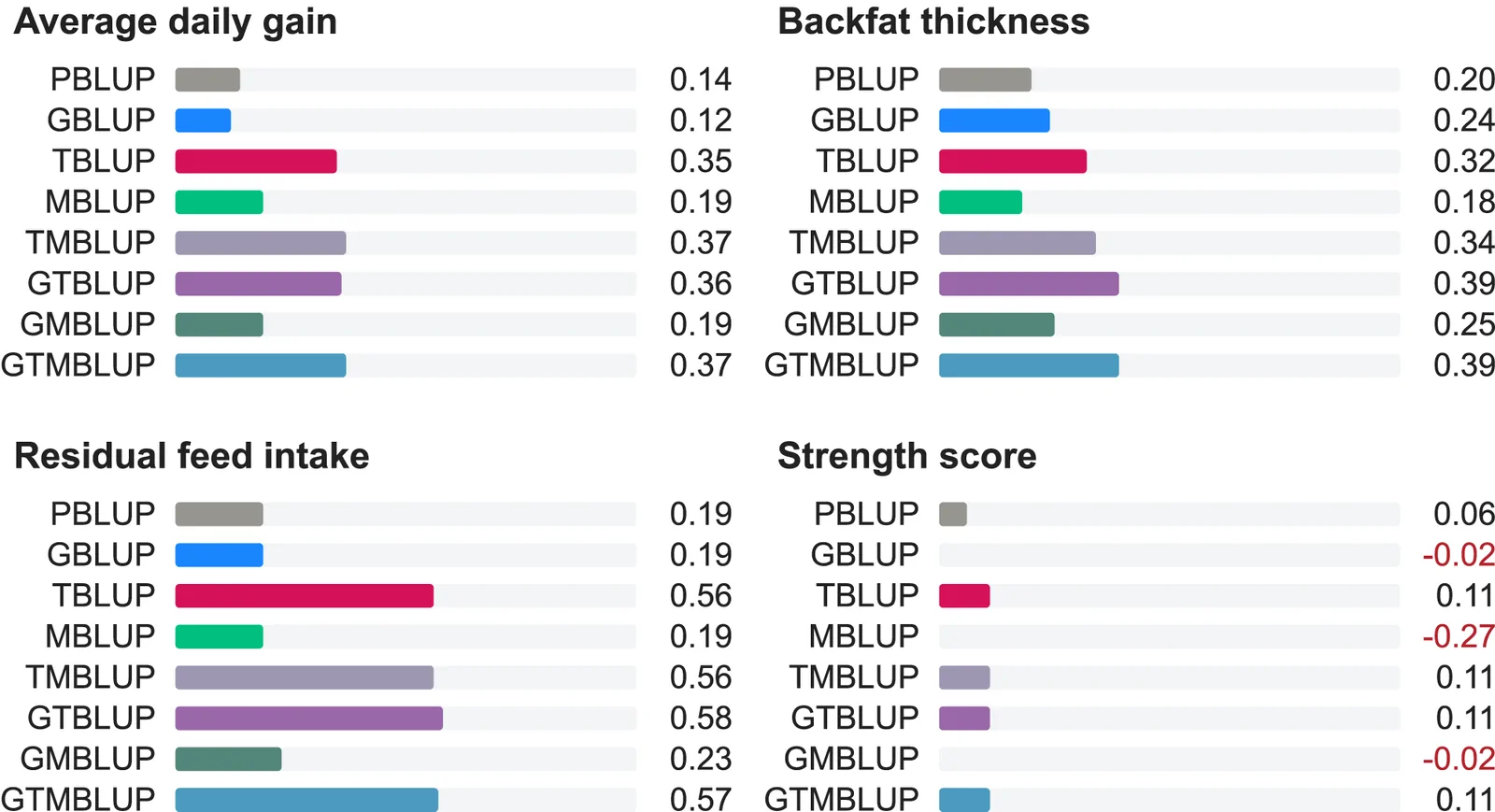
Phenotype prediction accuracies of the different models in leave-one-out cross-validation. Multi-breed scenario

We further assessed whether a reduced-density methylation panel could achieve prediction accuracies comparable to those of the full CpG set. To construct such a panel, we selected within each breed the 50,000 CpG sites with the largest standard deviation across samples. The intersection of these breed-specific sets yielded a set of 16,074 CpG sites common to all breeds. All models with methylomic effects were fitted using both the full set of 1,095,304 CpG sites (~1 M) and the reduced set of 16,074 CpG sites (~16k). Unless otherwise specified, the variance component estimates (Fig. 1; [Additional file 4, Figures S5–S8]; [Additional file 2, Table S9]) and prediction accuracies (Fig. 2; Table 1; [Additional file 1, Tables S1–S3]) presented in this article were obtained using the 16k CpG set.

**Table 1:** Phenotype prediction accuracies in leave-one-out cross-validation

Model	ADG	BFT	RFI	STR
PBLUP	0.14	0.20	0.19	0.06
GBLUP	0.12	0.24	0.19	−0.02
TBLUP	0.35	0.32	0.56	0.11
MBLUP	0.19	0.18	0.19	−0.27
TMBLUP	0.37	0.34	0.56	0.11
GTBLUP	0.36	0.39	0.58	0.11
GMBLUP	0.19	0.25	0.23	−0.02
GTMBLUP	0.37	0.39	0.57	0.11

ADG, average daily gain; BFT, backfat thickness; RFI, residual feed intake; STR, strength score

We used the DNA methylation levels to construct the methylation relationship matrix as

6 $$\mathbf{M} = \frac{\mathbf{C}\mathbf{C}^{\top}}{\mathrm{n}_{\mathrm{C}}},$$

where **C** is the matrix of methylation levels standardized per CpG site (centred to a mean of zero and scaled to unit variance) and n_C_ is the number of CpG sites.

### Statistical models

We analysed phenotypes using single-omics and multi-omics linear mixed models. The general model was:

7 $${\bf{y} = \bf{X}\bf{b} + \mathop \sum \limits_{\rm{i}} {\mathbb{I}_{\rm{i}}}{{\bf{Z}}_{\rm{i}}}{{\bf{u}}_{\rm{i}}} + \bf{e}},$$

where **y** is a vector of preadjusted phenotypes, **X** is an incidence matrix linking animals to fixed effects, **b** is a vector of fixed effects, $\mathbb{I}_{\mathrm{i}}$ is an indicator variable equal to 1 if the random effect **u**_i_ was included in the model (and 0 otherwise), **Z**_i_ is an incidence matrix linking animals to random effects, **u**_i_ is a vector of random animal effects associated with layer i (pedigree or omics), and **e** is a vector of residuals. Random effects were assumed to be mutually independent and multivariate normally distributed as $\mathbf{u}_{\mathrm{i}} \sim \mathcal{N}\left(\mathbf{0}, \mathbf{K}_{\mathrm{i}}{\sigma}_{\mathrm{i}}^2\right)$ and $\mathbf{e} \sim \mathcal{N}\left(\mathbf{0}, \mathbf{I}{\sigma}_{\mathrm{e}}^2\right)$, where **K**_i_ denotes the corresponding relationship matrix (pedigree **A**, genomic **G**, transcriptomic **T**, or methylomic **M**), **I** is an identity matrix, ${\sigma}_{\mathrm{i}}^2$ is the variance of the random effect **u**_i_, and ${\sigma}_{\mathrm{e}}^2$ is the error variance. We estimated the genetic (${\sigma}_{\mathrm{g}}^2$), transcriptomic (${\rm{\sigma }}_{\rm{t}}^2$), and methylomic (${\sigma}_{\mathrm{m}}^2$) variance components to quantify the contribution of each omics layer to phenotypic variation, along with the error variance (${\sigma}_{\mathrm{e}}^2$). These estimates were subsequently used in the mixed model equations to predict the random effects of the different omics layers for each animal. Variance components were estimated by restricted maximum likelihood (REML) [38, 39] using the ASReml-R package [40–42]. We note that variance components may have been affected by the fact that they were based on preadjusted phenotypes [43].

Single-omics models included only one omics source at a time, as indicated by the first letter of the model’s acronym. For example, the methylomic BLUP model (MBLUP) was defined as

8 $$\mathbf{y} = \mathbf{X}\mathbf{b} + \mathbf{Z}\mathbf{u}_{\mathrm{m}} + \mathbf{e},$$

with $\mathbf{e} \sim \mathcal{N}\left(\mathbf{0}, \mathbf{I}{\sigma}_{\mathrm{e}}^2\right)$. Multi-omics models (TMBLUP, GTBLUP, GMBLUP, and GTMBLUP) combined more than one omics layer, as indicated by their acronyms. For example, the GTMBLUP model incorporated genomic, transcriptomic, and methylomic random effects:

9 $$\mathbf{y} = \mathbf{X}\mathbf{b} + \mathbf{Z}\left(\mathbf{u}_{\mathrm{g}} + \mathbf{u}_{\mathrm{t}} + \mathbf{u}_{\mathrm{m}}\right) + \mathbf{e},$$

where $\mathbf{u}_{\mathrm{g}} \sim \mathcal{N}\left(\mathbf{0}, \mathbf{G}{\sigma}_{\mathrm{g}}^2\right)$, $\mathbf{u}_{\mathrm{t}} \sim \mathcal{N}\left(\mathbf{0}, \mathbf{T}{\sigma}_{\mathrm{t}}^2\right)$, $\mathbf{u}_{\mathrm{m}} \sim \mathcal{N}\left(\mathbf{0}, \mathbf{M}{\sigma}_{\mathrm{m}}^2\right)$, and $\mathbf{e} \sim \mathcal{N}\left(\mathbf{0}, \mathbf{I}{\sigma}_{\mathrm{e}}^2\right)$. The mixed model equations for GTMBLUP are then

10 $$\left\lbrack \begin{array}{cccc}\mathbf{X}^{\top}\mathbf{X} & \mathbf{X}^{\top}\mathbf{Z} & \mathbf{X}^{\top}\mathbf{Z} & \mathbf{X}^{\top}\mathbf{Z} \\\mathbf{Z}^{\top}\mathbf{X} & \mathbf{Z}^{\top}\mathbf{Z} + \mathbf{G}^{-1}\frac{\sigma_{\mathrm{e}}^2}{\sigma_{\mathrm{g}}^2} & \mathbf{Z}^{\top}\mathbf{Z} & \mathbf{Z}^{\top}\mathbf{Z} \\\mathbf{Z}^{\top}\mathbf{X} & \mathbf{Z}^{\top}\mathbf{Z} & \mathbf{Z}^{\top}\mathbf{Z} + \mathbf{T}^{-1}\frac{\sigma_{\mathrm{e}}^2}{\sigma_{\mathrm{t}}^2} & \mathbf{Z}^{\top}\mathbf{Z} \\\mathbf{Z}^{\top}\mathbf{X} & \mathbf{Z}^{\top}\mathbf{Z} & \mathbf{Z}^{\top}\mathbf{Z} & \mathbf{Z}^{\top}\mathbf{Z} + \mathbf{M}^{-1}\frac{\sigma_{\mathrm{e}}^2}{\sigma_{\mathrm{m}}^2}\end{array} \right\rbrack\left\lbrack \begin{array}{c} \hat{\mathbf{b}} \\ \hat{\mathbf{u}}_{\mathrm{g}} \\ \hat{\mathbf{u}}_{\mathrm{t}} \\ \hat{\mathbf{u}}_{\mathrm{m}} \end{array} \right\rbrack=\left\lbrack \begin{array}{c} \mathbf{X}^{\top}\mathbf{y} \\ \mathbf{Z}^{\top}\mathbf{y} \\ \mathbf{Z}^{\top}\mathbf{y} \\ \mathbf{Z}^{\top}\mathbf{y} \end{array} \right\rbrack$$

We calculated the proportion of phenotypic variance due to omics layer i as

11 $$\mathrm{c}_{\mathrm{i}}^2 = \frac{{\hat{\sigma}}_{\mathrm{i}}^2}{\sum_{\mathrm{j}} {\hat{\sigma}}_{\mathrm{j}}^2 + {\hat{\sigma}}_{\mathrm{e}}^2},$$

where ${\hat{\sigma}}_{\mathrm{i}}^2$ is the REML point estimate of the variance attributable to omics layer i, and the denominator is the sum of the residual variance and all other variance components j in the model.

### Prediction scenarios

We evaluated three prediction scenarios: within-breed, multi-breed, and across-breed. For each model, an animal’s predicted phenotype was the sum of the predicted random effects for the information sources in that model; in the multi-breed models, the estimated breed fixed effect was also added.

#### Within-breed predictions

In the within-breed scenario, we analysed each breed separately to test the models. We estimated variance components either from all data or within breeds. We predicted phenotypes using BLUP with leave-one-out (LOO) cross-validation, removing one animal at a time and predicting it from a model trained on all remaining animals of that breed. For prediction, we applied BLUP using variance components based on animals from all breeds combined.

#### Multi-breed predictions

In the multi-breed scenario, we jointly analysed animals from all three breeds, including breed as a fixed effect. We applied two cross-validation schemes: five-fold cross-validation and LOO cross-validation. In five-fold cross-validation, the dataset was partitioned into five folds using stratified random sampling by breed, ensuring that each fold contained approximately equal proportions of Duroc, Landrace, and Yorkshire animals; we then summarized the five resulting correlation coefficients by their median. The fold partitions were identical to those in Vourlaki et al. [44]. In LOO cross-validation, we removed one animal at a time and predicted its phenotype using a model trained on all remaining animals across breeds. In both schemes, we first estimated variance components using all animals, then applied BLUP to the validation animal or animals as if their phenotypes were unavailable, even though those phenotypes had contributed to the variance component estimation.

#### Across-breed predictions

In the across-breed scenario, we applied leave-one-breed-out (LOBO) cross-validation, in which we entirely excluded one breed during training and used it as an independent validation set. The variance components applied in BLUP were obtained from REML estimates using all breeds.

In all scenarios, we assessed prediction accuracy as the Pearson correlation between preadjusted phenotypes and their predicted values.

## Results

### Descriptive statistics

The adjusted means (standard deviations) were −6.44 (80.26) g/day for ADG, −0.04 (0.63) mm for BFT, −3.22 (131.25) g/day for RFI, and −0.16 (3.37) for STR (Table 2). Histograms of trait distributions are in [Additional file 3, Figures S1–S4]. Because the phenotypes were preadjusted for systematic effects using larger reference populations, their means in the analysed subset were close to, but not exactly, zero. Phenotypic correlations among traits are in Table 3.

**Table 2:** Descriptive statistics for the preadjusted phenotypes

Trait	n	Mean	SD	Min.	Max.
Average daily gain (g/day)	443	−6.44	80.26	−224.1	231.3
Backfat thickness (mm)	443	−0.04	0.63	−1.81	2.36
Residual feed intake (g/day)	443	−3.22	131.25	−889.6	525.0
Strength score	443	−0.16	3.37	−7.59	6.58

Preadjusted for systematic effects. n, number of animals; SD, standard deviation; Min., minimum; Max., maximum

**Table 3:** Correlations among preadjusted phenotypes

Trait	ADG	BFT	RFI
BFT	0.16		
RFI	0.17	0.33	
STR	−0.04	0.21	0.08

ADG, average daily gain; BFT, backfat thickness; RFI, residual feed intake; STR, strength score

### Variance component estimates

Figure 1 shows REML-based variance component estimates for the four traits, using animals from all breeds. Within-breed estimates are in [Additional file 4, Figures S5–S8], and numerical values of the variance proportion estimates and standard errors in the multi-breed scenario are in [Additional file 2, Table S9].

Table 4 shows pedigree-based heritability estimates for each trait. These estimates were derived from REML analyses that included only additive genetic effects, utilizing the numerator relationship matrix and animals from all breeds. STR exhibited the lowest heritability (0.16), while BFT and RFI had the highest heritabilities (0.42 and 0.39, respectively). Standard errors were relatively large, indicating uncertainty around the point estimates.

**Table 4:** Pedigree-based heritability estimates

Trait	**h**^2^	SE
ADG	0.28	0.15
BFT	0.42	0.14
RFI	0.39	0.14
STR	0.16	0.14

Variance components estimated using animals from all breeds. h^2^, heritability; SE, standard error

GBLUP produced variance component estimates largely similar to those of PBLUP, although a larger difference was observed for STR. In TBLUP, transcriptomic effects accounted for 0.68, 0.78, 1.00, and 0.09 of the phenotypic variance for ADG, BFT, RFI, and STR, respectively. The corresponding proportions attributed to methylation effects in MBLUP were 0.41, 0.41, 0.57, and 0.01. The methylomic component exceeded the genomic one for ADG and RFI, but not for BFT or STR, across the combined models. In GTBLUP, including transcriptomic effects reduced the proportion of variance attributed to genomic effects from 0.15 to 0.08 for ADG, from 0.31 to 0.23 for BFT, from 0.23 to 0.13 for RFI, and from 0.04 to 0.03 for STR, compared with GBLUP. For all traits, whenever transcriptomic effects were present in the model, they explained the largest variance among the omics components.

Across models, inclusion of additional omics effects reduced the proportion of residual variance, while redistributing the explained variance among the genomic, transcriptomic, and methylomic components. Several variance components were estimated to be zero, indicating that the REML solutions occurred at the boundary of the parameter space. Estimates from these models should therefore be interpreted with caution. For example, the residual variance for RFI was estimated to be zero in models that included transcriptomic effects. In TBLUP, this boundary solution left transcriptomic effects accounting for essentially all phenotypic variance. The within-breed analyses yielded a similar pattern of variance component partitioning [Additional file 4, Figures S5–S8].

The conformation trait STR stood out as the trait with the greatest residual variance across all models, with low heritability and modest contributions from the omics components. In contrast, RFI had lower residual variance proportions, with transcriptomic effects accounting for a large share of the total variance.

### Prediction accuracies in the multi-breed scenario

#### Leave-one-out cross-validation

Table 1 and Fig. 2 show LOO cross-validation prediction accuracies in the multi-breed scenario. In the multi-breed scenario, prediction accuracies were higher than in the within-breed analyses, consistent with a larger number of animals in the training data set. Accuracies differed across traits. Because smaller within-breed sample sizes can lead to less stable breed-specific estimates, the multi-breed scenario provides a more robust summary of overall prediction patterns.

Models including transcriptomic information (TBLUP, TMBLUP, GTBLUP, and GTMBLUP) consistently achieved the highest prediction accuracies. Prediction accuracies were highest for RFI and lowest for STR. Methylation-only models (MBLUP) showed moderate prediction accuracies for ADG, BFT, and RFI, but performed poorly for STR.

Compared with TBLUP, GTBLUP increased prediction accuracy from 0.35 to 0.36 for ADG, from 0.32 to 0.39 for BFT, and from 0.56 to 0.58 for RFI, whereas the accuracy for STR remained unchanged at 0.11. In contrast, adding methylation to transcriptomic models (TMBLUP vs. TBLUP, and GTMBLUP vs. GTBLUP) resulted in little or no change in prediction accuracy. As a result, the three-omics model (GTMBLUP) performed similarly to the best two-omics models.

#### Five-fold cross-validation

Results from the stratified five-fold cross-validation in the multi-breed scenario are in [Additional file 1, Table S1]. Overall, prediction accuracies were similar to those observed in the LOO cross-validation scheme and followed the same pattern, with transcriptomic models outperforming genomic and methylomic ones, and combined omics models often achieving the highest accuracies. The main difference was in the accuracies for STR, which were slightly higher than those from the LOO cross-validation scheme, with no negative values being present. Accuracies for the GTMBLUP model were 0.39, 0.39, 0.59, and 0.17 for ADG, BFT, RFI, and STR, respectively.

### Prediction accuracies within breeds

Within-breed prediction accuracies are in [Additional file 1, Table S2]. These were computed using REML variance components estimated from the full multi-breed dataset. Accuracies based on variance components estimated separately within each breed were also evaluated and gave similar results. Overall, within-breed accuracies showed patterns similar to the multi-breed analyses, but were generally lower, a consequence of the smaller sample sizes. Exceptions were the accuracies for ADG of the Landrace pigs, which were slightly higher than those from the multi-breed analyses.

### Prediction accuracies in the across-breed scenario

Prediction accuracies for the leave-one-breed-out (LOBO) cross-validations using approximately 16 thousand CpG sites are in [Additional file 1, Table S3]. Accuracies for models using the methylation relationship matrix derived from approximately 1 million CpG sites are in [Additional file 1, Table S7] and were in general similar to those obtained with the reduced 16k CpG panel.

Prediction accuracies from LOBO cross-validation different by breed and trait and were generally lower than those observed in the within-breed LOO cross-validations. Genomic models showed low accuracies, while transcriptomic and multi-omics models showed higher accuracies. The highest accuracies across all scenarios were attained by the TBLUP and GTBLUP models for RFI in LOBO.

### Single-omics vs. multi-omics

Aggregate averages of prediction accuracies across all traits, breeds, and cross-validation schemes are in [Additional file 1, Table S8]. Given the heterogeneity of the combinations, these values offer a broad overview of relative model performance but should be interpreted with caution.

Prediction accuracies differed across single-omics models, with TBLUP outperforming both GBLUP and MBLUP for all traits. When extending models by adding a second omics layer, the effect depended on the combination considered. Adding genomic information to transcriptomic models (GTBLUP) resulted in small changes relative to TBLUP, with slight increases for some traits and little difference for others. Adding methylation to genomic models (GMBLUP) resulted in a small average improvement in accuracy relative to GBLUP, though results varied by trait and prediction scenario. Extending models from two to three omics layers (GTMBLUP) resulted in only minor changes relative to GTBLUP, with both increases and decreases observed depending on the trait.

### Number of CpG sites evaluated in the model

For each model including methylomic effects, we evaluated two versions, using either 16,074 or 1,095,304 CpG sites ([Additional file 1, Tables S4–S7]). Overall, the 16k models showed more stable prediction performance across traits, model specifications, and cross-validation schemes, and in some instances outperformed the 1 M versions. For GTMBLUP, prediction accuracies were almost identical in the 16k and 1 M implementations.

### Summary of main findings

For models that included transcriptomic and methylomic profiles from longissimus muscle, transcriptomic effects explained the largest proportion of phenotypic variance and produced the highest prediction accuracies across traits, breeds, and cross-validation schemes. Although methylomic variance components were often larger than genomic components, this did not translate into superior predictive performance. TBLUP was the best-performing single-omics model and combining genomic and transcriptomic effects yielded the highest overall accuracies. This pattern is also reflected in the aggregate results ([Additional file 1, Table S8]), where transcriptomic and combined models showed the highest average prediction accuracies, while the inclusion of methylation resulted in smaller changes in accuracy.

## Discussion

To our knowledge, this is the first study in animal breeding to jointly use genomic, epigenomic, and transcriptomic profiles measured on the same individuals to predict complex traits. In this paper, we employed linear mixed models for phenotype prediction. In the companion paper by Vourlaki et al. [44], deep learning methods were applied on the same dataset.

Studies incorporating DNA methylation directly into genomic or trait prediction have been conducted in plant species, including *Arabidopsis thaliana* [26, 45, 46] and barley (*Hordeum vulgare*) [47, 48], and on humans, where methylation data have been used to predict traits such as biological age, smoking, alcohol intake, blood cholesterol, body mass index, height, clinical depression, and rheumatoid arthritis [49–55]. By using cytosine methylation profiles from RRBS, our study extends this line of inquiry to livestock, where multi-omics prediction has begun to be explored [24, 29, 30, 56–69].

Another line of inquiry involves using epigenomic and transcriptomic data to preselect or assign weights to SNPs [70–73]. However, these omics measurements are typically derived from animals outside the prediction dataset, rather than being measured in the prediction animals themselves.

### Transcriptomic vs. epigenomic contributions

Among the omics layers evaluated from longissimus muscle biopsies, transcriptomic information accounted for 0.68, 0.78, 1.00, and 0.09 of the phenotypic variance for ADG, BFT, RFI, and STR, respectively. Compared with GBLUP, TBLUP increased phenotype prediction accuracy by 0.23 for ADG, 0.08 for BFT, 0.37 for RFI, and 0.13 for STR. The higher prediction accuracies obtained with transcriptomic information for ADG, BFT, and RFI agree with previous studies reporting improved phenotype prediction when gene-expression data were incorporated [29, 64, 66, 67, 74–78]. However, there have also been studies in which transcriptomic models did not outperform genomic models. For example, Xu et al. [79] reported superior performance of genomic models over transcriptomic models in maize, while Li et al. [80] found no improvement from incorporating gene expression data in *Drosophila melanogaster*. These discrepancies likely reflect differences in biological context rather than a general limitation of transcriptomic data. In livestock, the predictive value of gene expression depends on tissue relevance and how closely the timing of sample collection corresponds to the period of trait expression, as previously observed in mice [78] and pigs [29, 67].

In contrast, DNA methylation explained a smaller proportion of phenotypic variance and yielded only modest gains in predictive accuracy. While methylation generally underperformed as a standalone predictor, it occasionally provided incremental improvements when combined with other omics layers. The stronger predictive performance of transcriptomic data likely reflects its closer functional proximity to the phenotype, as gene expression represents a downstream cellular state that arises from the combined effects of genetic and regulatory influences. Moreover, DNA methylation is only one component of cellular regulation, alongside transcription factors, chromatin remodelling, and histone modifications, and may therefore explain only part of the variation observed at the transcriptomic level.

### Effect of the number of CpG sites

Reducing the number of CpG sites from approximately 1 million to approximately 16 thousand had model- and trait-dependent effects on predictive performance. In methylomic-only models (MBLUP), the reduced CpG set (16k) consistently gave higher prediction accuracies across traits, breeds, and cross-validation schemes ([Additional file 1, Tables S4–S7]), with the largest differences observed for STR, the trait with the lowest heritability. Models with transcriptomic information (TMBLUP and GTMBLUP) showed almost identical results for both CpG sets, whereas GMBLUP showed small and inconsistent differences. The high correlation between off-diagonal elements of the methylation relationship matrices from the full and reduced CpG sets (*r* = 0.91) indicates that the smaller set captured most of the similarity among animals. A similar pattern has been observed in genomic prediction using whole-genome sequence data, where increasing marker density beyond a certain threshold provides little improvement in prediction accuracy [81], largely due to redundancy and linkage disequilibrium between markers.

### Modelling

The models applied in this study are based on the standard BLUP framework [82–85], with additional molecular sources of variation (omics) fitted as independent random effects. For example, in GTMBLUP, genomic, transcriptomic, and methylomic effects were included as separate random effects, whose sum determines the predicted phenotype. The covariance structures for the genomic, transcriptomic, and methylomic components were defined using relationship matrices proportional to **WW**^⊤^, where **W** represents a standardized omics matrix. This framework preserves the covariance structure of conventional quantitative genetic models and enables evaluation of the relative contributions of each omics-derived relationship matrix to predictive performance.

However, one limitation should be noted. The models we applied assume independence between the genomic, transcriptomic, and methylomic effects, although these layers are biologically connected. Genetic variants can affect both methylation [86, 87] and gene expression [88, 89], and DNA methylation can influence transcription [34, 90]. Associations among SNPs, CpG sites, and transcripts have been reported [91], indicating that the independence assumption is a simplification of the truth.

More flexible approaches have been proposed to relax these model assumptions. For example, CORE GREML [92] allows covariances between random effects, while multi-omics BLUP extensions, such as GTCBLUP [64, 78], aim to account for shared or redundant information between predictors. Another alternative is genomics–omics BLUP (GOBLUP) [63, 93–97], which adopts a recursive framework in which one model relates the phenotype to genomic and omics predictors, while a second model relates omics predictors to genomic information. Beyond linear mixed models, non-linear approaches such as deep learning may offer greater flexibility for phenotype prediction, as explored in the companion study by Vourlaki et al. [44].

From a breeding perspective, interest typically lies in predicting breeding values rather than phenotypes. One approach would be to rely only on genomic breeding values from models such as GBLUP, GTBLUP, GMBLUP, or GTMBLUP. However, this raises concerns about confounding, as genetic and omics effects are not independent.

### Within-breed vs. across-breed prediction

Across-breed (LOBO) prediction accuracies were sometimes comparable to, or higher than, within-breed accuracies, despite the greater genetic distance between the training and validation populations. This indicates that prediction was portable across breeds, although the larger training population used for LOBO prediction may also have improved accuracy. Duroc had the lowest average LOBO accuracy, consistent with the closer genetic relationship between the Landrace and Yorkshire breeds.

### Relevance to breeding and selection

In animal breeding, the main quantity of interest is the breeding value, because only heritable genetic effects are expected to be transmitted stably to the next generation through inherited haplotypes. In this study, however, we evaluated the prediction of phenotypes rather than breeding values. This distinction is important because improved phenotypic prediction from epigenomic or transcriptomic information does not imply that the variation captured by these omics data is heritable.

Transcriptomic information, for example, may improve phenotypic prediction because gene expression is biologically closer to the phenotype. Such an improvement does not necessarily indicate improved prediction of genetic merit. Genomic information differs fundamentally from transcriptomic and epigenomic measurements in this respect. The genotype is inherited and is not affected by the environment, whereas gene expression and DNA methylation are influenced by both genetic and environmental factors. If the phenotype of a validation individual y_0_ is decomposed as $\mathrm{y}_0 = \mathrm{a}_0 + \mathrm{e}_0$, where $\mathrm{a}_0$ is the additive genetic effect and $\mathrm{e}_0$ is the environmental effect, an EBV ${\hat{\textrm{a}}}_0$ based on inherited genetic information is expected to satisfy

12 $$\mathrm{Cov}\left({\hat{\textrm{a}}}_0, \mathrm{e}_0\right) = 0,$$

such that

13 $$\mathrm{Cov}\left({\hat{\textrm{a}}}_0, \mathrm{y}_0\right) = \mathrm{Cov}\left({\hat{\textrm{a}}}_0, \mathrm{a}_0\right) + \mathrm{Cov}\left({\hat{\textrm{a}}}_0, \mathrm{e}_0\right) = \mathrm{Cov}\left({\hat{\textrm{a}}}_0, \mathrm{a}_0\right).$$

For a predictor ${\hat{\textrm{u}}}_{0,{\mathrm{omics}}}$ based on transcriptomic or methylomic information, however, $\mathrm{Cov}\left({\hat{\textrm{u}}}_{0,\mathrm{omics}}, \mathrm{e}_0\right)$ may not be zero. Thus, high accuracy in predicting phenotypes from these omics measurements may partly reflect the capture of non-heritable variation and therefore does not necessarily translate into a greater response to selection.

In the linear mixed models fitted here, breeding values could in principle be predicted based on the genomic random effects of models such as GTBLUP, GMBLUP, or GTMBLUP, without adding the estimated epigenomic or transcriptomic effects to the prediction. Such predictions would differ from those obtained from a standard GBLUP model because the genomic effects in the multi-omics models are estimated jointly with the other omics effects. This raises the possibility that including omics terms could alter EBV accuracy by changing how variation is partitioned among genomic and non-genomic components. Ayres et al. [98] showed, in a single-locus setting, that omitting DNA methylation from the model does not bias breeding value estimates, indicating that omics information is not required for unbiased estimation of breeding values under the assumptions of that model. However, the consequences for EBV accuracy were not evaluated.

In genome-wide models, epigenomic and transcriptomic effects may partly capture genetic signal that is imperfectly represented by the SNP panel. Conversely, because DNA methylation and gene expression are themselves partly genetically regulated, omics effects may absorb variation that would otherwise be attributed to the genomic component. This complicates variance partitioning and limits the interpretation of estimated omics components as effects independent of the genome. From a selection perspective, the relevant issue is whether omics information improves prediction of genetic merit, either by helping to separate additive genetic effects from non-genetic sources of variation [22] or by providing information about genetic effects not adequately captured by the available SNP genotypes. Addressing this question would require validation against an independent measure of genetic merit, such as progeny performance, which is beyond the scope of this study.

Implementation considerations are also important when using omics information for breeding and selection. Transcriptomic and epigenomic measurements are more costly than genotyping and depend on the tissue, technique, and time of sampling, making the choice of where and when to measure them an additional component of the prediction problem.

Nevertheless, phenotypic prediction has practical relevance in livestock production. It may support management and precision livestock farming, particularly for repeated or longitudinal traits of economic importance. Semen quality in boars or bulls is one example, as it is repeatedly assessed throughout a sire’s reproductive life and is itself commercially relevant. In such cases, accurate phenotype prediction can be useful even when predictions partly reflect non-heritable effects.

### Limitations and further work

The number of animals within individual breeds was not very large, especially considering the complexity of multi-omics models, which may contribute to uncertainty in the within-breed estimates of variance components and prediction accuracies. Increasing sample sizes would allow more robust comparisons between models and clearer assessment of contributions from each omics layer. In addition, variance components used in the cross-validation analyses were estimated from the complete dataset. This constitutes a mild form of data leakage that may lead to some overestimation of prediction accuracy.

Furthermore, the modelling framework assumed independence between omics layers, which may not fully capture biological dependencies. More flexible approaches that explicitly model interactions between molecular layers, such as GOBLUP [93, 96, 97] and CORE GREML [92], can be explored in future work.

The omics layers considered in this study were genomics, transcriptomics, and epigenomics (DNA methylation). Additional molecular layers, such as chromatin accessibility, histone modifications, proteomics, and metabolomics, may provide complementary predictive information and could be explored in future work. Within the methylomics layer, CpG methylation levels could alternatively be modelled using regional averages to reduce noise at individual sites. Broader DNA methylation assays, such as whole-genome bisulphite sequencing (WGBS) [99], may capture signals from CpG sites not assayed by RRBS. Alternative methods, such as methylated DNA immunoprecipitation (MeDIP) [100–102] and nanopore sequencing [103–105], could also be explored.

Lastly, transcriptomic and methylomic data were obtained from the longissimus muscle. Given the tissue-specific nature of gene expression and epigenetic regulation, the predictive value of these molecular layers may depend on how closely the sampled tissue reflects the biology of each trait. It is reasonable to attribute part of the strong transcriptomic performance observed to the functional relevance of this tissue for the traits examined, as well as to the proximity between the time of sampling and the period of phenotypic expression. Profiling additional tissues would help assess the generality of these findings and could identify tissues with greater predictive ability for specific traits.

## Conclusions

In summary, including epigenomic (RRBS) and transcriptomic (RNA-seq) profiles from longissimus dorsi muscle biopsies enhanced predictive performance relative to models based solely on genomic information. Transcriptomic and methylomic components captured additional variance beyond pedigree and genomic effects. In particular, transcriptomic data consistently yielded the largest gains in accuracy. In contrast, DNA methylation contributed more modest, trait- and breed-dependent improvements. Notably, reducing the dimensionality of methylation data by selecting a subset of highly variable CpG sites did not compromise predictive performance, as models based on approximately 16 thousand sites performed comparably to those using the set with approximately 1 million sites.

At the same time, the benefits of incorporating additional molecular layers were not uniform, and gains in prediction accuracy depended on the trait, breed, and modelling context considered. Our results show that integrating multiple sources of molecular information can improve phenotype prediction in livestock populations, but they also indicate that the inclusion of additional omics does not automatically translate into substantial gains in accuracy.

## Electronic supplementary material

Below is the link to the electronic supplementary material.

Supplementary Material 1: Tables S1 to S8

Supplementary Material 2: Table S9

Supplementary Material 3: Figures S1 to S4

Supplementary Material 4: Figures S5 to S8

## Data Availability

The datasets analysed in this study are not publicly available due to confidentiality agreements with commercial partners.
