# Supplementary material for: Multi-omics prediction of swine production traits with linear mixed models
Source: Genet Sel Evol. 2026 Nov 10;58:84. doi: 10.1186/s12711-026-01100-6 (PMC13753436; doi:10.1186/s12711-026-01100-6)
Supplement: Supplementary file 1 [file 12711_2026_1100_MOESM1_ESM.pdf]

Supplementary Information

Tables of phenotype prediction accuracies

Table S1 Median phenotype prediction accuracies across folds in a five-fold cross-validation using all breeds

Model	ADG	BFT	RFI	STR
PBLUP	0.18	0.18	0.21	0.11
GBLUP	0.20	0.24	0.18	0.00
TBLUP	0.39	0.33	0.58	0.17
MBLUP	0.20	0.19	0.14	0.05
TMBLUP	0.38	0.37	0.58	0.17
GTBLUP	0.36	0.39	0.60	0.17
GMBLUP	0.16	0.26	0.19	0.06
GTMBLUP	0.39	0.39	0.59	0.17

Table S2 Within-breed phenotype prediction accuracies (leave-one-out cross-validation)

Model	Duroc				Landrace				Yorkshire			
	ADG	BFT	RFI	STR	ADG	BFT	RFI	STR	ADG	BFT	RFI	STR
PBLUP	0.07	0.01	0.24	0.05	0.19	0.36	0.08	−0.12	0.01	−0.01	0.19	0.06
GBLUP	0.07	0.06	0.14	−0.14	0.21	0.35	0.13	−0.37	−0.10	0.11	0.25	−0.12
TBLUP	0.29	0.29	0.45	0.18	0.46	0.33	0.56	−0.07	0.36	0.13	0.45	−0.07
MBLUP	0.14	0.03	0.29	−0.37	0.21	0.34	0.15	−0.80	−0.05	0.03	0.14	−0.84
TMBLUP	0.33	0.25	0.47	0.18	0.48	0.34	0.54	−0.07	0.34	0.13	0.49	−0.07
GTBLUP	0.31	0.30	0.46	0.19	0.47	0.39	0.56	−0.09	0.34	0.18	0.49	−0.04
GMBLUP	0.13	0.07	0.26	−0.14	0.22	0.36	0.17	−0.37	−0.05	0.12	0.23	−0.12
GTMBLUP	0.33	0.30	0.46	0.19	0.48	0.39	0.55	−0.09	0.33	0.18	0.51	−0.04

Table S3 Across-breed phenotype prediction accuracies (leave-one-breed-out cross-validation)

Model	Duroc				Landrace				Yorkshire			
	ADG	BFT	RFI	STR	ADG	BFT	RFI	STR	ADG	BFT	RFI	STR
GBLUP	0.01	0.05	0.11	0.06	0.03	0.22	0.00	0.05	−0.08	0.16	0.08	−0.03
TBLUP	0.14	0.24	0.50	0.16	0.33	0.45	0.66	0.03	0.31	0.27	0.52	0.18
MBLUP	0.14	0.02	0.16	−0.05	0.06	0.09	−0.01	−0.09	0.18	0.07	0.02	0.14
TMBLUP	0.16	0.23	0.50	0.16	0.32	0.45	0.62	0.03	0.33	0.28	0.49	0.18
GTBLUP	0.14	0.24	0.50	0.17	0.33	0.49	0.66	0.04	0.29	0.31	0.52	0.17
GMBLUP	0.13	0.10	0.18	0.06	0.06	0.20	0.00	0.05	0.14	0.17	0.05	−0.03
GTMBLUP	0.16	0.24	0.50	0.17	0.32	0.49	0.63	0.04	0.32	0.31	0.50	0.17

Table S4 Multi-breed phenotype prediction accuracies (leave-one-out cross-validation): 16k vs. 1M CpGs

Model		ADG	BFT	RFI	STR
MBLUP	16k	0.19	0.18	0.19	−0.27
	1M	0.08	0.04	0.12	−0.94
TMBLUP	16k	0.37	0.34	0.56	0.11
	1M	0.36	0.32	0.55	0.11
GMBLUP	16k	0.19	0.25	0.23	−0.02
	1M	0.14	0.26	0.22	−0.02
GTMBLUP	16k	0.37	0.39	0.58	0.11
	1M	0.37	0.39	0.57	0.11

16k, 16,074 CpG sites; 1M, 1,095,304 CpG sites.

Table S5 Median phenotype prediction accuracies from multi-breed five-fold cross-validation: 16k vs. 1M CpGs

Model		ADG	BFT	RFI	STR
MBLUP	16k	0.20	0.19	0.14	0.05
	1M	0.12	0.08	0.07	0.01
TMBLUP	16k	0.38	0.37	0.58	0.17
	1M	0.38	0.33	0.57	0.17
GMBLUP	16k	0.16	0.26	0.19	0.06
	1M	0.15	0.27	0.20	0.06
GTMBLUP	16k	0.39	0.39	0.59	0.17
	1M	0.37	0.39	0.59	0.17

16k, 16,074 CpG sites; 1M, 1,095,304 CpG sites.

Table S6 Within-breed phenotype prediction accuracies (leave-one-out cross-validation): 16k vs. 1M CpGs

Model		Duroc				Landrace				Yorkshire			
		ADG	BFT	RFI	STR	ADG	BFT	RFI	STR	ADG	BFT	RFI	STR
MBLUP	16k	0.14	0.02	0.16	−0.05	0.06	0.09	−0.01	−0.09	0.18	0.07	0.02	0.14
	1M	0.05	0.15	0.15	−0.02	0.08	0.07	0.03	−0.09	0.13	0.07	0.02	0.09
TMBLUP	16k	0.16	0.23	0.50	0.16	0.32	0.45	0.62	0.03	0.33	0.28	0.49	0.18
	1M	0.15	0.24	0.49	0.16	0.33	0.45	0.65	0.03	0.32	0.27	0.50	0.18
GMBLUP	16k	0.13	0.10	0.18	0.06	0.06	0.20	0.00	0.05	0.14	0.17	0.05	−0.03
	1M	0.04	0.11	0.18	0.06	0.06	0.23	0.03	0.05	−0.03	0.18	0.06	−0.03
GTMBLUP	16k	0.16	0.24	0.50	0.17	0.32	0.49	0.63	0.04	0.32	0.31	0.50	0.17
	1M	0.14	0.23	0.50	0.17	0.33	0.49	0.65	0.04	0.31	0.31	0.51	0.17

16k, 16,074 CpG sites; 1M, 1,095,304 CpG sites.

Table S7 Across-breed phenotype prediction accuracies (leave-one-breed-out cross-validation): 16k vs. 1M CpGs

Model		Duroc				Landrace				Yorkshire			
		ADG	BFT	RFI	STR	ADG	BFT	RFI	STR	ADG	BFT	RFI	STR
MBLUP	16k	0.17	−0.07	0.31	0.09	0.21	0.35	0.12	*	−0.42	0.05	0.14	−0.03
	1M	0.01	−0.01	−0.01	−0.38	*	*	*	−0.48	−0.14	−0.23	0.07	*
TMBLUP	16k	0.33	0.23	0.46	0.21	0.48	0.34	0.56	*	0.35	0.08	0.49	−0.03
	1M	0.27	0.23	0.44	0.19	0.40	0.32	0.56	−0.48	0.35	−0.03	0.48	*
GMBLUP	16k	0.17	0.07	0.31	0.10	0.22	0.36	0.15	*	−0.50	0.10	0.24	0.03
	1M	0.07	0.09	0.18	0.06	0.19	0.36	0.14	−0.48	−0.14	0.11	0.26	0.03
GTMBLUP	16k	0.33	0.23	0.46	0.19	0.48	0.40	0.57	*	0.35	0.13	0.51	0.03
	1M	0.29	0.23	0.44	0.19	0.47	0.40	0.56	−0.48	0.35	0.13	0.50	0.03

16k, 16,074 CpG sites; 1M, 1,095,304 CpG sites.
* Indicates convergence problems.

Table S8 Aggregate mean phenotype prediction accuracies across models and traits

Model	Average accuracy
PBLUP	0.08
GBLUP	0.09
TBLUP	0.30
MBLUP	0.03
TMBLUP	0.31
GTBLUP	0.32
GMBLUP	0.10
GTMBLUP	0.32

Values are means across different traits and heterogeneous model types and should be interpreted with caution.
