# Supplementary material for: Multi-omics prediction of swine production traits with linear mixed models
Source: Genet Sel Evol. 2026 Nov 10;58:84. doi: 10.1186/s12711-026-01100-6 (PMC13753436; doi:10.1186/s12711-026-01100-6)
Supplement: Supplementary file 2 [file 12711_2026_1100_MOESM2_ESM.pdf]

Table S9 Variance component proportion estimates and standard errors (multi-breed)

Model	Component	ADG		BFT		STR		RFI	
		c^2	SE	c^2	SE	c^2	SE	c^2	SE
PBLUP	G	0.28	(0.15)	0.42	(0.14)	0.16	(0.14)	0.39	(0.14)
	e	0.72	(0.15)	0.58	(0.14)	0.84	(0.14)	0.61	(0.14)
GBLUP	G	0.15	(0.08)	0.31	(0.09)	0.04	(0.07)	0.23	(0.09)
	e	0.85	(0.08)	0.69	(0.09)	0.96	(0.07)	0.77	(0.09)
TBLUP	T	0.68	(0.19)	0.78	(0.19)	0.09	(0.07)	1.00	(0.00)
	e	0.32	(0.19)	0.22	(0.19)	0.92	(0.07)	0.00	(0.00)
MBLUP	M	0.41	(0.15)	0.41	(0.15)	0.01	(0.08)	0.57	(0.16)
	e	0.59	(0.15)	0.59	(0.15)	0.99	(0.08)	0.43	(0.16)
TMBLUP	T	0.67	(0.19)	0.77	(0.19)	0.09	(0.07)	0.78	(0.10)
	M	0.25	(0.13)	0.21	(0.12)	0.00	(0.00)	0.22	(0.10)
	e	0.08	(0.20)	0.02	(0.20)	0.92	(0.07)	0.00	(0.00)
GTBLUP	G	0.08	(0.07)	0.23	(0.07)	0.03	(0.07)	0.13	(0.06)
	T	0.65	(0.19)	0.76	(0.19)	0.08	(0.07)	0.87	(0.06)
	e	0.28	(0.19)	0.01	(0.17)	0.89	(0.10)	0.00	(0.00)
GMBLUP	G	0.06	(0.08)	0.29	(0.10)	0.04	(0.07)	0.16	(0.09)
	M	0.34	(0.17)	0.16	(0.15)	0.00	(0.00)	0.43	(0.17)
	e	0.60	(0.16)	0.55	(0.15)	0.96	(0.07)	0.41	(0.16)
GTMBLUP	G	0.04	(0.06)	0.23	(0.07)	0.03	(0.07)	0.10	(0.07)
	T	0.66	(0.19)	0.76	(0.19)	0.08	(0.07)	0.78	(0.10)
	M	0.22	(0.13)	0.00	(0.00)	0.00	(0.00)	0.13	(0.10)
	e	0.08	(0.20)	0.01	(0.17)	0.89	(0.10)	0.00	(0.00)
