# Supplementary material for: Multi-omics prediction of swine production traits with linear mixed models
Source: Genet Sel Evol. 2026 Nov 10;58:84. doi: 10.1186/s12711-026-01100-6 (PMC13753436; doi:10.1186/s12711-026-01100-6)
Supplement: Supplementary file 3 [file 12711_2026_1100_MOESM3_ESM.pdf]

Histograms of adjusted phenotypes

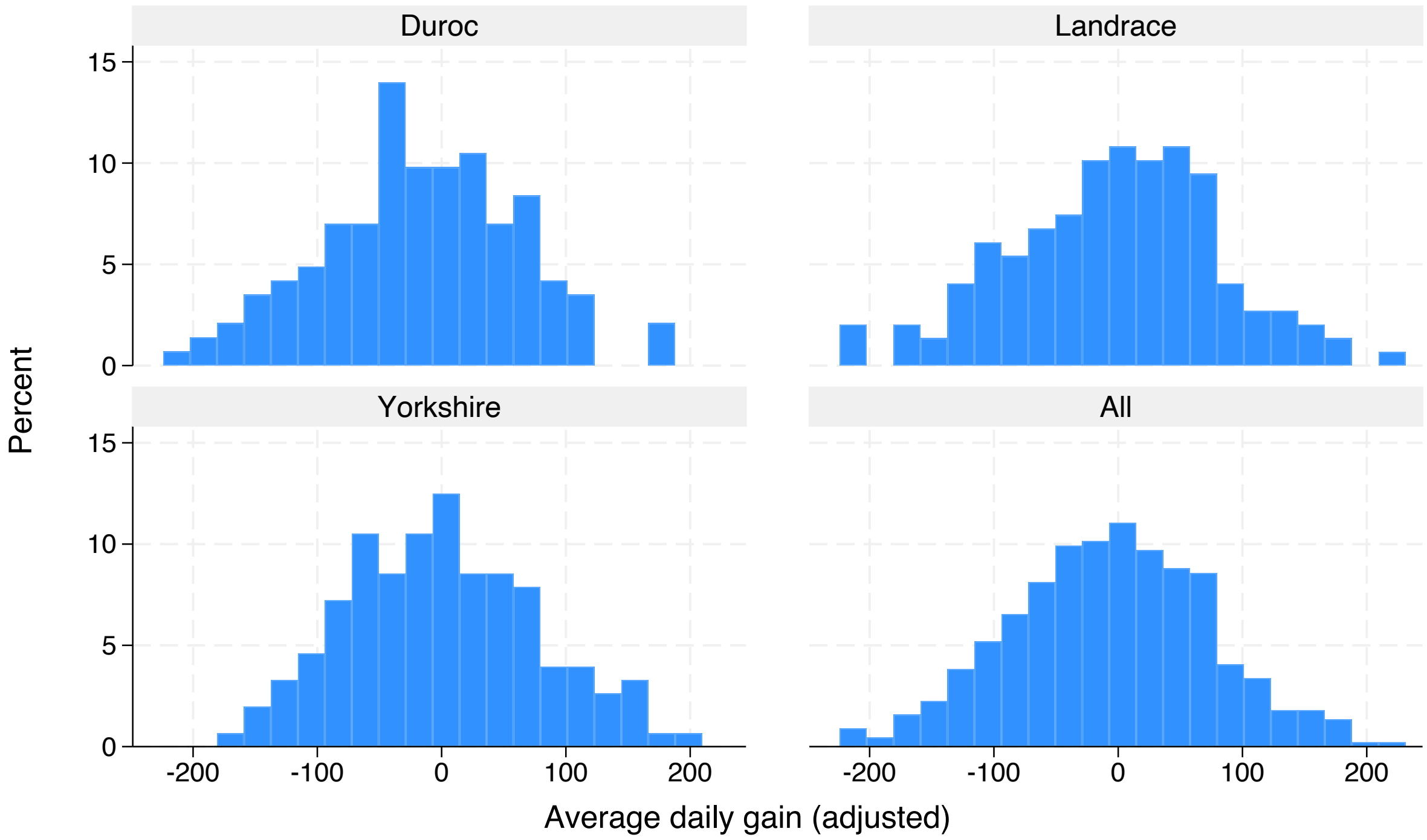

Fig. S1 Histograms of distributions of adjusted average daily gain, per breed and combined.

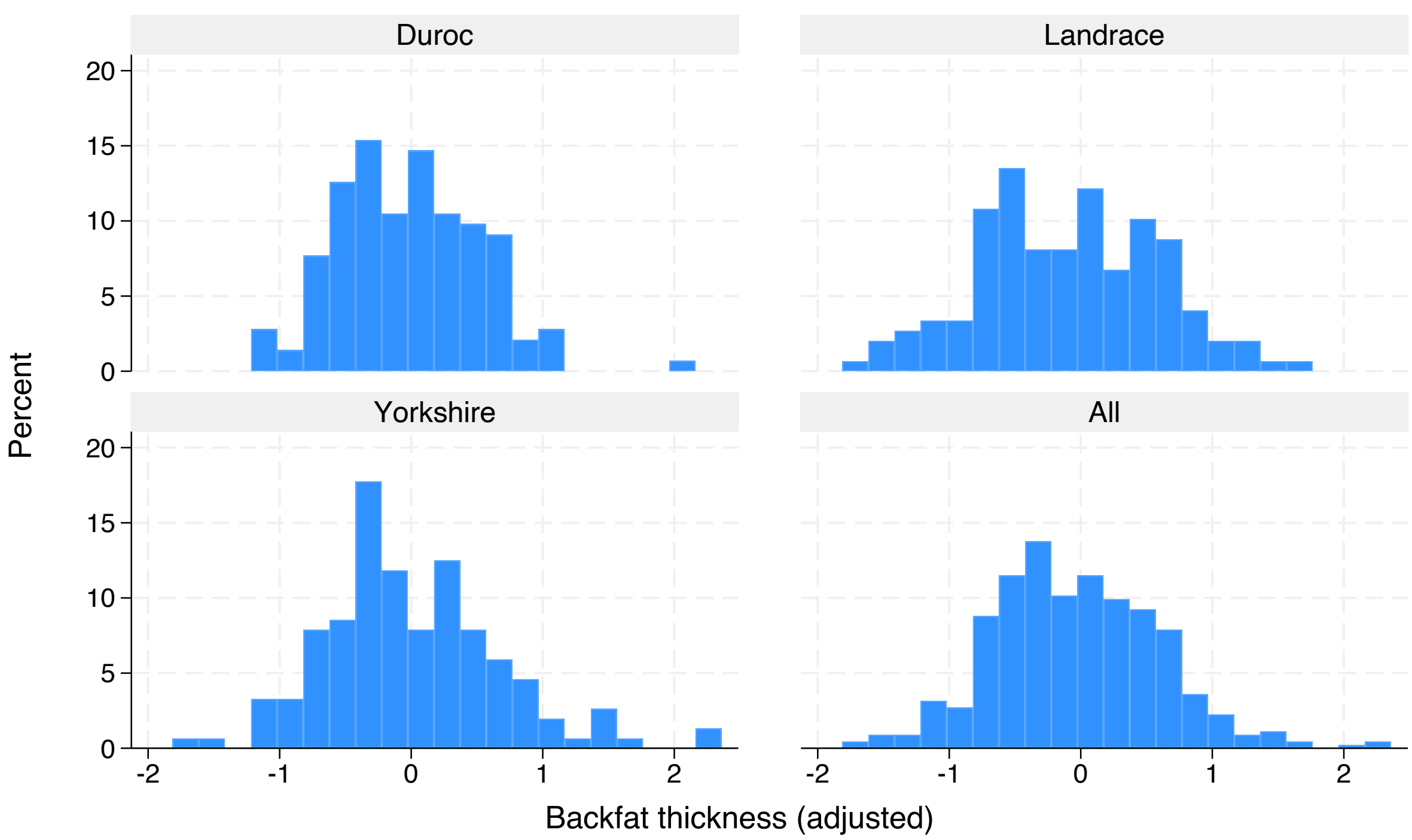

Fig. S2 Histograms of distributions of adjusted backfat thickness, per breed and combined.

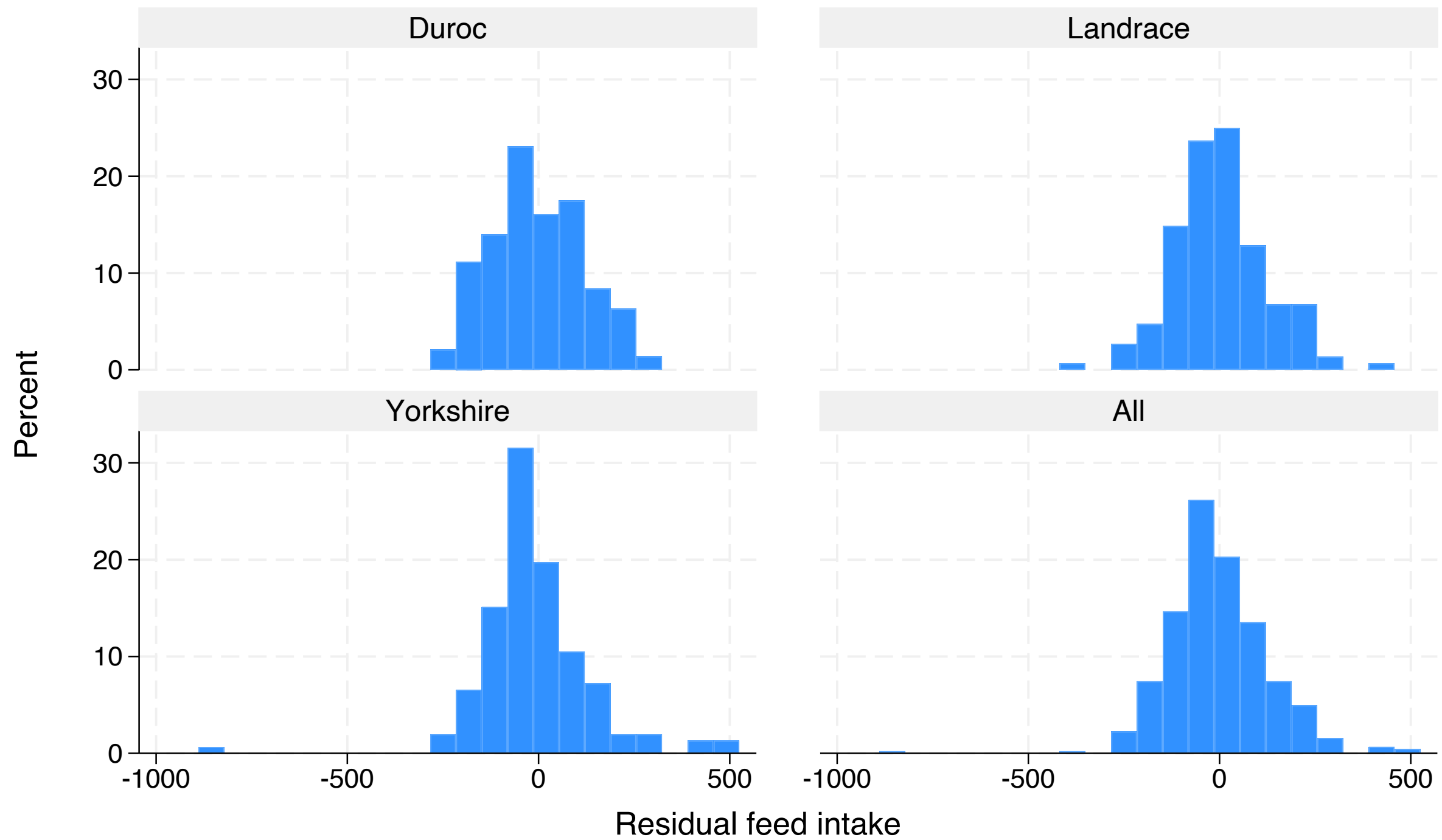

Fig. S3 Histograms of distributions of residual feed intake, per breed and combined.

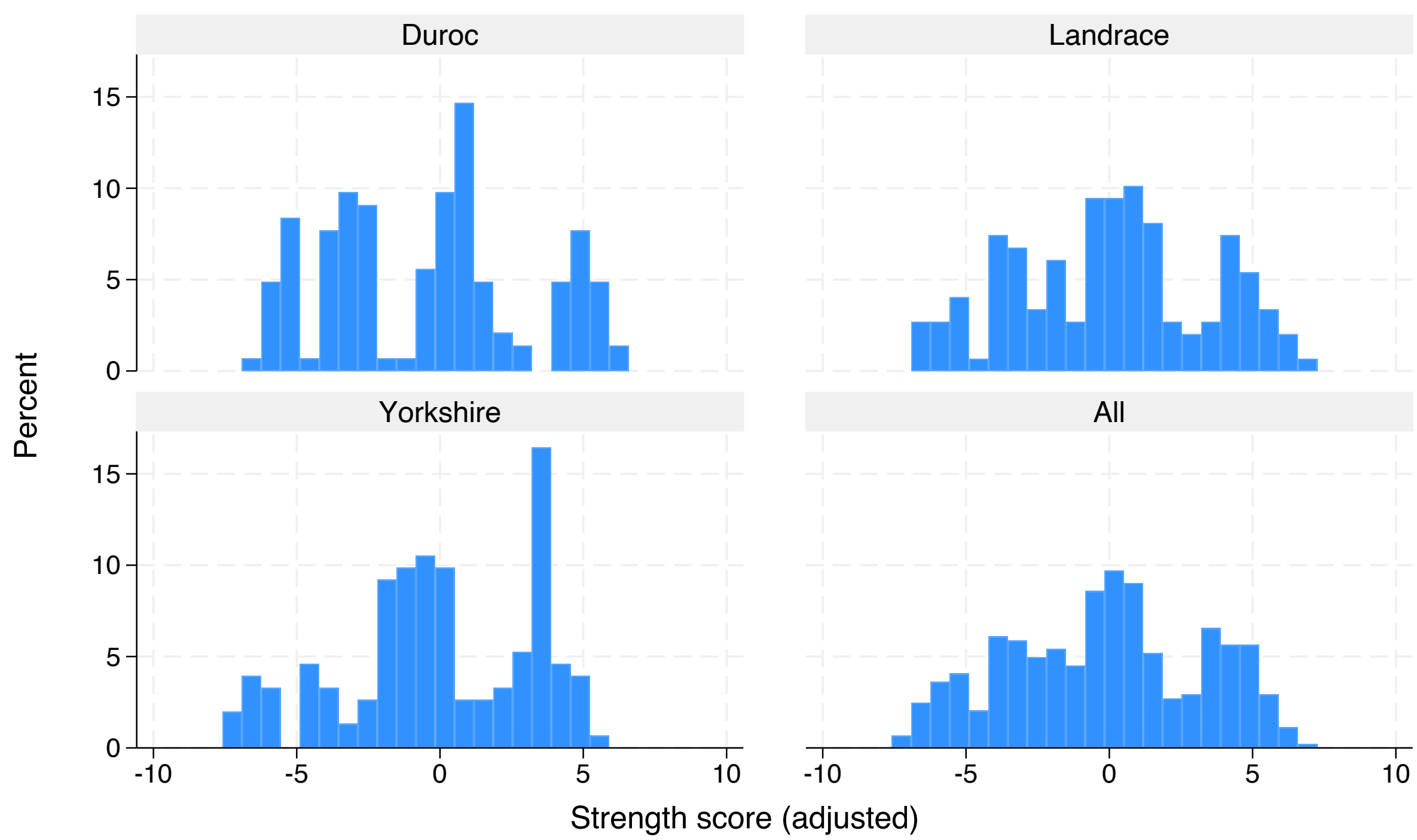

Fig. S4 Histograms of distributions of the adjusted trait strength score, per breed and combined.
