# Supplementary material for: Multi-omics prediction of swine production traits with linear mixed models
Source: Genet Sel Evol. 2026 Nov 10;58:84. doi: 10.1186/s12711-026-01100-6 (PMC13753436; doi:10.1186/s12711-026-01100-6)
Supplement: Supplementary file 4 [file 12711_2026_1100_MOESM4_ESM.pdf]

Variance partitioning bar graphs

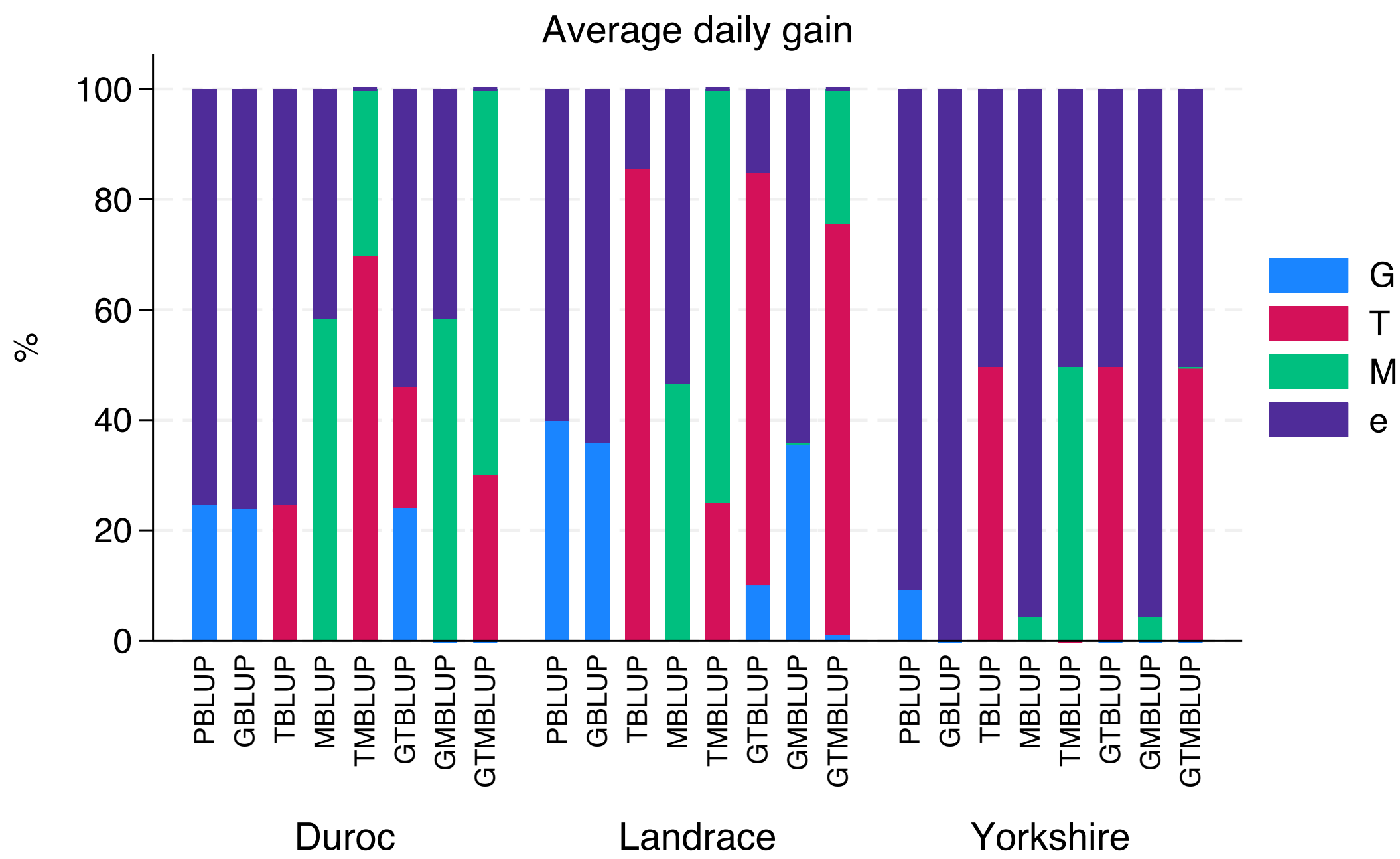

Fig. S5 Variance components expressed as proportions of the total phenotypic variance for average daily gain, based on REML estimates within breeds (Duroc, Landrace, and Yorkshire). Stacked bars represent the proportion of phenotypic variance attributed to each component within a given model. G: genomic effects; T: transcriptomic effects; M: methylomic effects; e: residual effects.

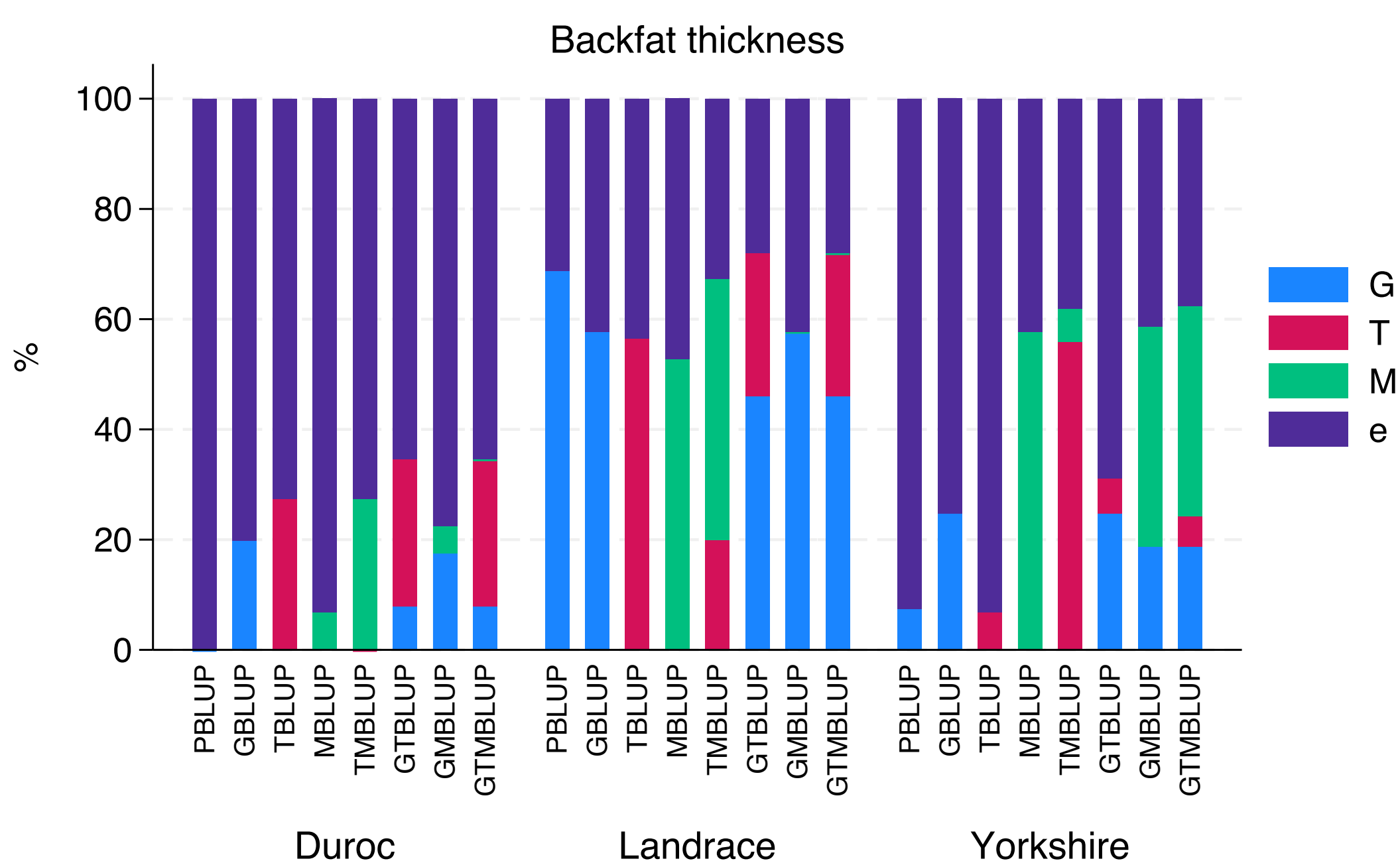

Fig. S6 Variance components expressed as proportions of the total phenotypic variance for backfat thickness, based on REML estimates within breeds.

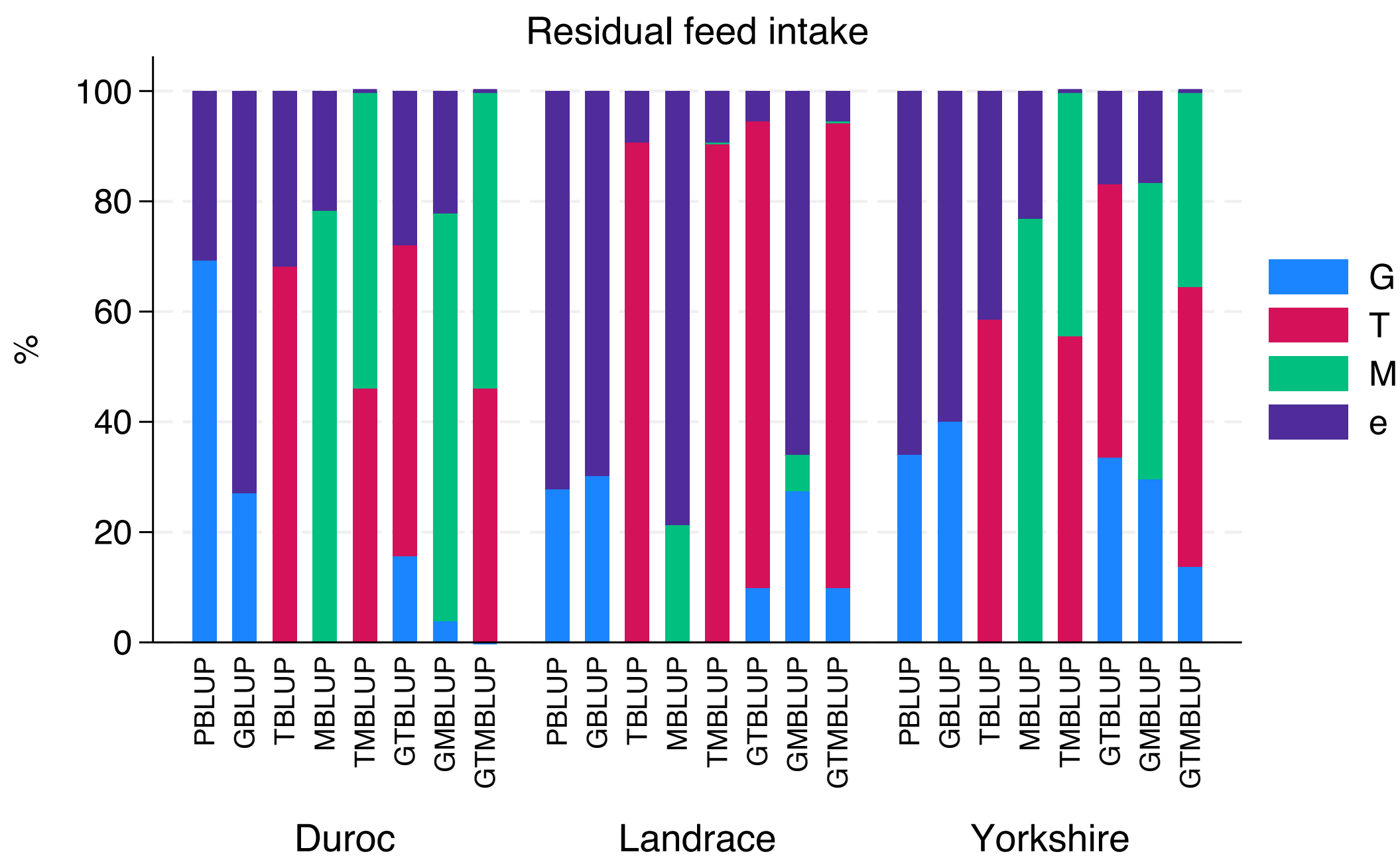

Fig. S7 Variance components expressed as proportions of the total phenotypic variance for residual feed intake, based on REML estimates within breeds.

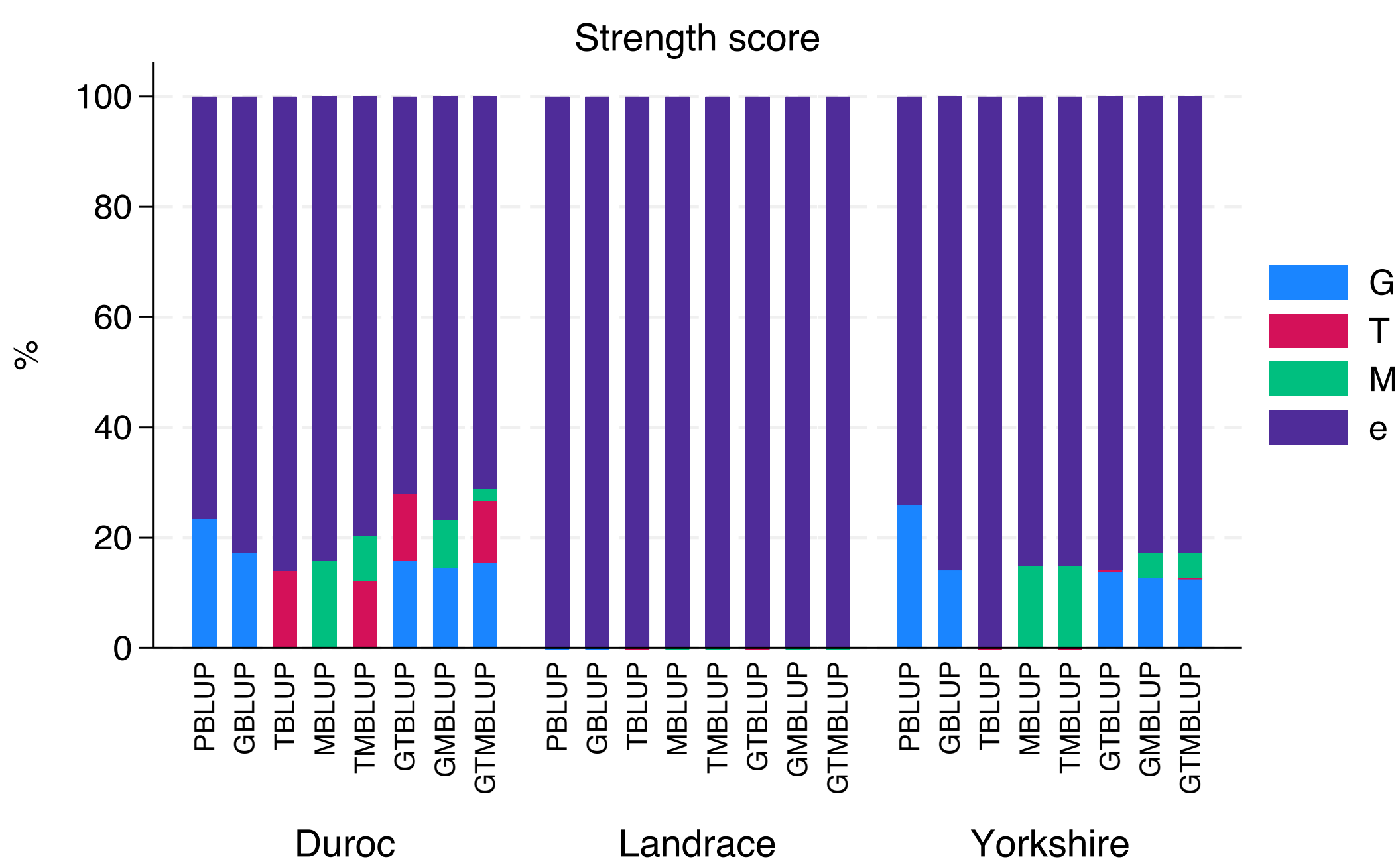

Fig. S8 Variance components expressed as proportions of the total phenotypic variance for strength score, based on REML estimates within breeds.
